# Early damage enhances compensatory responses to herbivory in wild lima bean

**DOI:** 10.3389/fpls.2022.1037047

**Published:** 2022-11-29

**Authors:** Carlos Bustos-Segura, Raúl González-Salas, Betty Benrey

**Affiliations:** Laboratory of Evolutionary Entomology, Institute of Biology, University of Neuchâtel, Neuchâtel, Switzerland

**Keywords:** multitrophic interactions, ontogeny, tolerance, defense induction, defense priming, timing of herbivory, seed interactions

## Abstract

Damage by herbivores can induce various defensive responses. Induced resistance comprises traits that can reduced the damage, while compensatory responses reduce the negative effects of damage on plant fitness. Timing of damage may be essential in determining the patterns of induced defenses. Here, we tested how timing and frequency of leaf damage affect compensatory responses in wild lima bean plants in terms of growth and seed output, as well as their effects on induced resistance to seed beetles. To this end, we applied mechanical damage to plants at different ontogenetical stages, at one time point (juvenile stage only) or two time points (seedling and juvenile stage or juvenile and reproductive stage). We found that plants damaged at the seedling/juvenile stage showed higher compensatory growth, and seed output compared to plants damaged only at the juvenile stage or juvenile/reproductive stage. Seeds from plants damaged at the juvenile and juvenile/reproductive stages had fewer beetles than seeds from undamaged plants, however this was driven by a density dependent effect of seed abundance rather than a direct effect of damage treatments. We did not find differences in parasitism rate by parasitoid wasps on seed beetles among plant treatments. Our results show that damage at the seedling stage triggers compensatory responses which implies that tolerance to herbivory is enhanced or primed by early damage. Herbivory often occurs at several time points throughout plant development and this study illustrates that, for a full understanding of the factors associated with plant induced responses in a dynamic biotic environment, it is important to determine the multitrophic consequences of damage at more than one ontogenetical stage.

## Introduction

The nature and magnitude of plant induced responses depend on several factors, such as the type, frequency and timing of damage and herbivore identity (Karban, 2011; Schuman and Baldwin, 2016). Herbivore damage can occur at several times throughout a plant’s life, during the same ontogenetical stage or at different stages. However, it is still not fully understood how the timing and frequency of herbivore damage affect the plant’s induced defense responses. Induced plant responses to damage comprise plastic traits that help plants increasing their fitness in the presence of natural enemies (Karban and Baldwin, 1997; Baldwin and Preston, 1999). Such responses can be classified as two different strategies, induced resistance and tolerance. Induced resistance includes inducible traits that protect plants from future attacks (Karban, 2011), such as direct defenses, both chemical (e.g. secondary metabolites) and physical (trichomes) defenses, and indirect defenses (volatile compounds and extrafloral nectar), that aid in the recruitment of natural enemies of herbivores (Gatehouse, 2002; Heil and Baldwin, 2002; Turlings and Erb, 2018).

Tolerance to herbivory refers to the plant's response after herbivory to reduce the negative effects of damage on fitness (Strauss and Agrawal, 1999; Núñez-Farfán et al., 2007) and it is the result of compensatory responses which can often be measured as compensatory growth and/or seed production (the ability of a plant to increase its biomass or reproduction after suffering damage). As tolerance involves changes in resource allocation that occur as a response to damage, it is also considered an induced defense (Karban and Myers, 1989). Because resistance and tolerance can be induced by herbivore damage, the time at which damage occurs determines the expression of both strategies. Yet it is still unclear the extent to which the timing of multiple damage events influences the expression of each strategy.

Plants present ontogenetic trajectories consisting of changes in the production and investment of defenses that occur across the developmental stages of the plant (Boege and Marquis, 2005; Barton and Boege, 2017). They are influenced by abiotic factors, type of defense, and/or amount and timing of herbivory. For example, in *Casearia nitida* it was shown that plants damaged at the sapling or reproductive stage compensate for defoliation by growing more new leaves than undamaged plants (Boege, 2005). However, this response varied with the amount of defoliation, since saplings compensated better than reproductive plants at high defoliation levels. The interactions between plants and a dynamic community of antagonists (herbivores) and mutualists (pollinators and natural enemies of herbivores) will be fundamental in shaping the ontogenetical patterns of defensive strategies. In this context, plants can use different strategies depending on the stage at which they interact with other species (Barton and Boege, 2017). For example, plants that suffer a single damage event would favor investment in compensatory responses, while plants that experience repeated damage could favor a higher investment in induced resistance (Karban et al., 1999). Ontogenetic trajectories for tolerance can also be species-specific. Barton (2013) found that tolerance mechanisms were different between two *Plantago* species depending on their ontogeny. While tolerance in *P. lanceolata* was associated with flowering and shoot biomass in plants damaged at a mature stage, in *P. major* it was associated more with biomass and photosynthetic parameters when damaged at seedling and juveniles stages, respectively. Finally, plant resistance and tolerance may have different trajectories. This could also indicate trade-offs between both strategies if the expression of one strategy over the other switches according to the ontogenetical stage. For example, it has been shown that in *Raphanus sativus* juvenile plants show higher resistance but less tolerance than reproductive plants (Boege et al., 2007), but in *Arabidopsis thaliana* the cost of tolerance at different stages was not associated with a trade-off with resistance (Kornelsen and Avila-Sakar, 2015).

Plant resistance and tolerance responses to herbivore damage can cascade to other interactions that occur later during the plant’s life. Rusman et al. (2020) studied the interaction between the timing of herbivore damage on flower traits and the pollinator community in *Brasica nigra*. They found that plants attacked at vegetative stages by some herbivore species had lower number of flowers, fewer flower visitations by pollinators and lower seed output than control undamaged plants, while damage at the budding stage had positive effects on flower visitation. Similarly, previous work with wild lima bean, showed that damage by leaf beetles reduced subsequent attack by seed beetles at a later stage of the plant’s life (Abdala-Roberts et al., 2016; Hernández-Cumplido et al., 2016; Bustos-Segura et al., 2020). However, this type of induction depends on herbivore species and on the plant’s stage when the first damage occurs (in leaves or bean pods) (Hernández-Cumplido et al., 2016). In another study, Cuny et al. (2018) found that when lima bean plants were exposed to a single herbivory event by leaf caterpillars, plants produced more leaves compared to undamaged plants, indicating leaf overcompensation. The results from the studies above provide strong evidence that induced responses in wild lima bean are dependent on several biotic factors, however the extent to which the frequency and time of damage influence these responses has not been tested. Particularly, it remains unknown whether early leaf damage events can modify or even improve tolerance to herbivory or affect seed quality and preferences of seed feeders and their predators or parasitoids. Analyses of ontogenetic interactions, could provide insights into how ontogenetical trajectories influence defense induction for future interactions in a multitrophic context.

Here we used wild lima bean to test the effects of frequency of damage and damage at different ontogenetic stages on compensatory responses and subsequent interactions with seed insects. To represent better the frequency and timing of herbivory as it occurs in the wild, we applied mechanical damage at two time points representing early damage (at seedling + juvenile stages) or late damage (at the juvenile + reproductive stages). In addition, to analyze the influence of one vs. two damage events at different ontogenetical stages on tolerance and induced resistance, another group of plants was damaged only at the juvenile stage. This design allowed us to test differences in timing (seedling vs. reproductive) and the synergistic effects of different events of damage (one vs two events of damage) that comprise the span of the plant’s development. Once seeds were matured, we examined the effects of leaf damage on seed infestation by exposing seeds to natural populations of seed beetles and their parasitoids. We addressed the following questions: 1) How does the timing and frequency of leaf damage influence plant growth and reproductive output? 2) How does timing and frequency of leaf damage alter the subsequent interactions between seeds and their associated insects? In addition, because we expected that a damage event early in the plant’s life could determine the investment in tolerance to damage at future stages, we tested the hypothesis that damage at the seedling stage will enhance plant tolerance, compared to damage at the juvenile stage or repeated damage at the juvenile and adult stages.

## Material and methods

### Study system

Wild lima bean (*Phaseolus lunatus*) is distributed along the Pacific coast from Mexico to South America (Freytag and Debouck, 2002). In the south pacific Mexican coast this annual legume germinates in June–July, produces flowers in October–November and seeds in December–January (Heil and Silva Bueno, 2007; Moreira et al., 2015; Hernández-Cumplido et al., 2016). During the growing stage, lima bean plants are attacked by a number of leaf-chewing insects, including the velvet armyworm *Spodoptera latifascia*, Lepidoptera: Noctuidae (Cuny et al., 2018). This polyphagous caterpillar attacks several crops including maize, beans, tomato and chili pepper (Habib et al., 1982; Chabaane et al., 2022). During seed production, which lasts around two months, seed beetle species such as *Zabrotes subfasciatus* (Coleoptera: Chrysomelidae) and *Acanthoscelides obtectus* (Coleoptera: Chrysomelidae) enter dry pods and lay eggs on lima bean seeds, the larvae develop and pupate inside the seed (Benrey et al., 1998; Alvarez et al., 2005; Šešlija et al., 2009). They are both considered insect pests that also attack other species of stored cultivated beans (Birch et al., 1985; Paul et al., 2009). Both species are parasitized by several solitary parasitoid species, among them, the ectoparasitoid *Stenocorse bruchivora* (Aebi et al., 2008; Moreira et al., 2015; Hernández-Cumplido et al., 2016).

### Plants

To explore the effects of timing of herbivory on plant responses, we conducted an experiment at the field station of the Universidad del Mar, Campus Puerto Escondido during the field season 2019-2020. We planted three seeds of wild lima bean in each of 80 pots (5 l volume) by November 15th, 2019. Seeds were collected in the same site the year before. After germination, we thinned the seedlings to leave only one per pot. Pots were kept in a large tent (Lumite^®^ 3.66 m × 1.83 m × 1.83 m, Bioquip, CA, USA) to avoid unwanted insects attacking the plants. After two weeks of germination, we selected 64 seedlings (with only one trifolia developed) and distributed them randomly in 16 mesh tents (four plants per tent; Lumite^®^ 1.83 m × 1.83 m × 1.83 m, Bioquip, CA, USA). Then, we applied damage treatments on these plants with the objective of studying induced plant responses to damage produced at different times throughout plant development, at seedling, juvenile or reproductive stage.

### Experimental procedure

**Figure 1:**
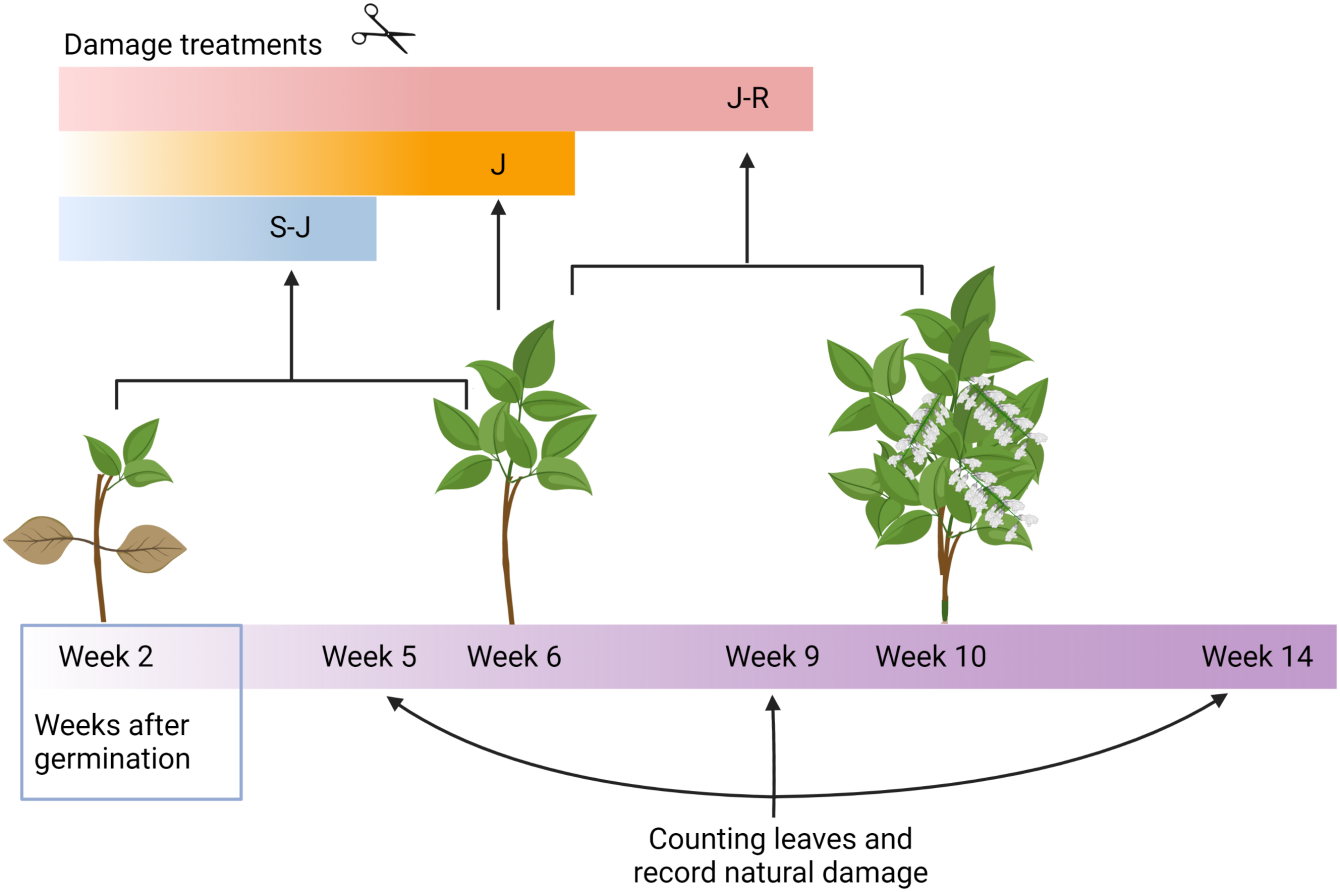
Schematic representation of the experimental design. Damage treatments on lima bean plants consisted of artificial damage on leaves at two different ontogenetical stages (S-J and J-R) or at one (J).

Four treatments were randomly assigned to the four plants in each of the 16 tents. The treatments to one plant per tent were as follows: C: control plants with no artificial damage. S-J: Plants damaged at the seedling and juvenile stages (at two and six weeks after germination). J: Plants damaged only at the juvenile stage (at six weeks after germination). J-R: plants damaged at the juvenile and reproductive stages (at six and ten weeks after germination; see **Figure 1**). So that all tents contained one plant of each treatment. For each damaged plant, we cut half of each leaflet with scissors. In addition, we also removed the apical meristem of growing tendrils. This was based on the damage that common herbivores, such as *Spodoptera latifascia*, produce on wild plants, as they would readily eat both the meristems and leaves (Bustos-Segura, personal observation).

### Plant measurements and collection of seed insects

We counted the number of leaves and branches per plant three times throughout plant development (5, 9 and 14 weeks after germination). As the mesh tent was not 100% effective at keeping out naturally occurring insect, we observed some damage on the plants (mainly by *Spodoptera* spp. and *Diabrotica baleata*). Thus, throughout the experiment we recorded the proportion of damaged leaves. We also recorded the date when each plant produced the first flower to calculate the time to flowering (days from germination to the production of the first flower).

When pods were mature, they were removed from the experimental plants and placed inside a single green mesh bag per plant, next to each plant following the methods from Bustos-Segura et al. (2020). This mesh was wide enough to allow the entrance of seed beetles and their parasitoids, but small enough to hold seeds inside the bags. Twelve weeks after germination, when most of the seeds were collected in mesh bags, the tent was removed to allow natural infestation by seed insects (including seed beetles and their parasitoids). After two weeks of exposure, the seeds were placed in plastic bags for two weeks to allow the emergence of insects developing inside the seeds. Then seeds were placed in a freezer at -20°C for one day. Insects found in each bag were counted and identified. We also counted the number of seeds and weighed 10 healthy seeds per plant.

### Statistical methods

All analyses were performed with R system (version 4.2.0; R Core Team, 2022). We used generalized linear mixed models (GLMM) to analyze differences among damage treatments on plant and insect variables using package *lme4* (Bates et al., 2015). For all models we included damage treatment as a fixed effect and tent as a random effect. For the number of leaves and natural damage we included time point of measurement and the interaction with treatment as another fixed effect, and plant ID as a random effect. For analyzing number of leaves, number of branches, number of seeds, and number of insects, a Poisson error distribution was used. For analyzing the number of seed insects we included the number of seeds (log-transformed) as a covariate to account for the density-dependent relationship between insects and seeds. As seed beetles are attacked by solitary parasitoids, the number of total seed insects (beetles plus parasitoids) indicates the initial infestation by seed beetles. Whenever the dispersion ration of Poisson models was higher than 2, we included an observation level factor as a random factor to control for overdispersion. For comparing natural damage among treatments we used a binomial error distribution (as the proportion of damage leaflets). Time to first flowering was analyzed with a Cox proportional hazards mixed model, with package *coxme*. Seed mass in seeds was analyzed with a normal error distribution. Differences in parasitism rate among treatments were analyzed with a GLMM and a binomial distribution. We performed a structural equation model (SEM) for testing the causal pathway among Treatment groups, number of trifolia (at the last measurement), number of seeds and number of insects. Parasitism rate was not included in the SEM, given it could only be estimated for 30 plants. We used the piecewiseSEM package in R system that allows to include GLMs and mixed models in a SEM (Lefcheck, 2016).

## Results

**Figure 2:**
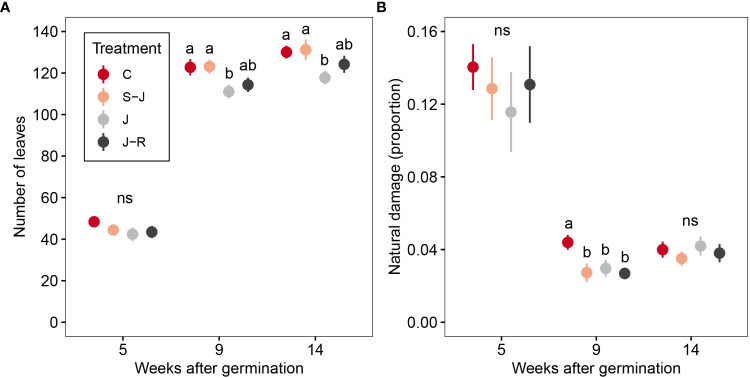
Effects of timing and frequency of damage on lima bean leaves. **(A)** number of leaves, **(B)** proportion of leaflets with uncontrolled natural herbivory. Treatment groups were: mechanically undamaged control plants (C); plants damaged at the seedling and juvenile stage (S-J), only at the juvenile stage or at the juvenile stage and reproductive stage (J-R). Different letters indicate significant differences among damage treatments within the same time point. ns indicate non-significant difference among damage treatments.

Overall, there was a significant effect of damage treatment on the number of trifolia across the season ( $\chi_{\left(3\right)}^{2}$ =10.63; P=0.014, **Figure 2A**), with only plants from the J treatment having significantly fewer leaves than control plants (**Table S1**). The effect sizes of the different contrasts ranged in the lower values (Cohen’s d from -0.04 to 0.11, **Table S1**). Time significantly explained the change in number of trifolia ( $\chi_{\left(2\right)}^{2}$ =2452; P<0.0001), meanwhile, the interaction between treatment and time was not statistically significant ( $\chi_{\left(6\right)}^{2}$ =3.15; P=0.79).The number of branches increased with time ( $\chi_{\left(2\right)}^{2}$ =170; P<0.0001; **Figure S1A**), but damage treatment and its interaction with time had a non-significant effect ( $\chi_{\left(3\right)}^{2}$ =6.26; P=0.1; $\chi_{\left(6\right)}^{2}$ =1.72; P=0.94, respectively). Despite the plants being inside tents, some insects entered and caused damage. For this minor but uncontrolled damage, there were differences across time ( $\chi_{\left(2\right)}^{2}$ =1115; P<0.0001), with higher damage at the beginning of the season (**Figure 2B**), but treatment as a main factor did not explain much of the variation ( $\chi_{\left(3\right)}^{2}$ =5.72; P=0.13). There was however, an effect of the interaction between treatment and time ( $\chi_{\left(6\right)}^{2}$ =29.38; P<0.0001), where in the middle of the season undamaged control plants received more natural damage than mechanically damaged plants (regardless of the timing of damage).This effect was small compared to the treatment damage and was not detected at the beginning, nor at the end of the season (**Figure 2B**).

The time from germination to first flowering was affected by the damage treatment ( $\chi_{\left(3\right)}^{2}$ =7.81; P=0.05). Plants from damage treatments S-J showed the longest time to production of the first flower and control plants the shortest time (**Figure S1B**). However, a multiple comparison posthoc test did not reveal specific differences between treatment pairs.

**Figure 3:**
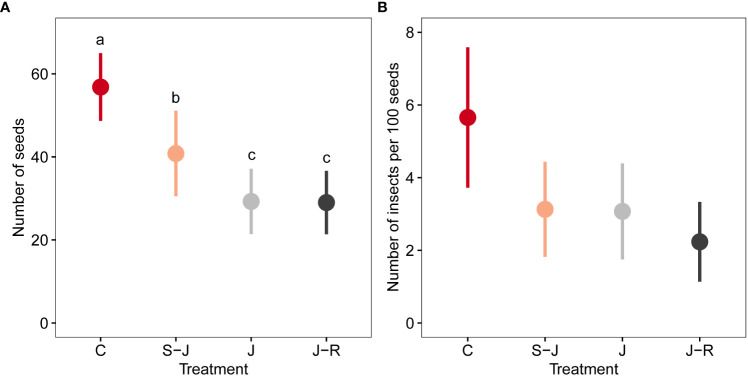
Effects of timing and frequency of damage on seed production and interactions with seed insects. **(A)** number of seeds as a measure of reproductive output and **(B)** number of seed insects per 100 seeds per plant. The number of insects includes seed beetles and parasitoids, thus indicating the initial infestation by seed beetles. Treatment groups were: mechanically undamaged control plants (C); plants damaged at the seedling and juvenile stage (S-J), only at the juvenile stage or at the juvenile stage and reproductive stage (J-R). Different letters indicate significant differences among damage treatments.

Fifty-two plants out of 64 produced seeds, but this was not influenced by the damage treatment ( $\chi_{\left(3\right)}^{2}$ =1.17; P=0.76). There was a significant effect of damage treatment on seed production ( $\chi_{\left(3\right)}^{2}$ =217; P<0.0001; **Figure 3A**). Control plants produced the most seeds, followed by plants damaged at the seedling stage (S-J), and then J and J-R plants. J and J-R plants produced similar number of seeds (**Table S1**). The effect sizes for comparisons between controls and damage treatments were relatively high (**Table S1**), while the effect sizes among damage treatments were lower (**Table S1**). Seed mass was not significantly different among treatments ( $\chi_{\left(3\right)}^{2}$ =5.55; P=0.14).

**Figure 4:**
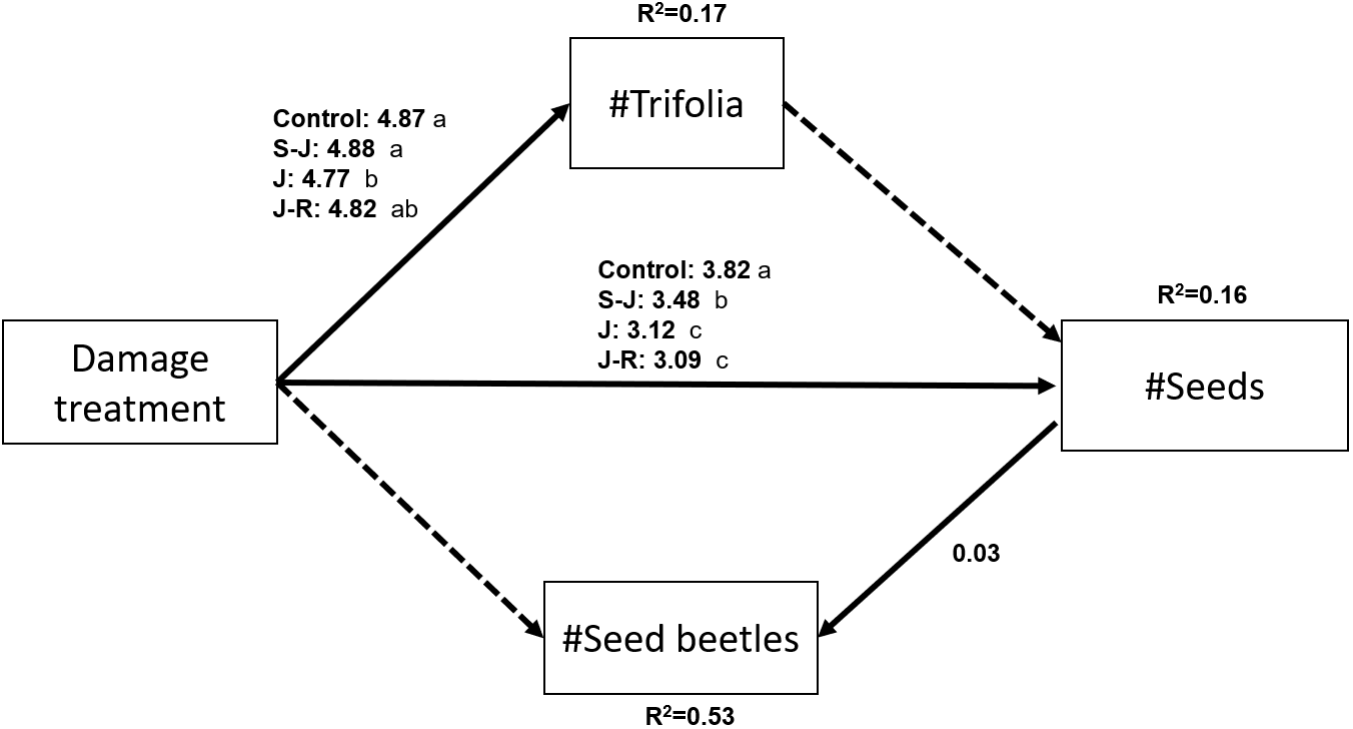
Diagram of the structural equation model including pathways between measured variables. Solid arrows indicate statistically significant positive pathways and dashed arrows indicate no significant pathways. The differences between treatments are given next to pathways from the Damage treatment variable and different letters indicate significant differences between groups according to a Tukey’s post-hoc test. R square values given for each individual endogenous variables are conditional r squares (excluding random effects). All pathways were analyzed with GLMs and a Poisson error distribution. The estimates are given in the log scale. Model goodness-of-fit: Fisher’s C=0.25, P=0.88.

The total number of beetles and parasitoids collected was 87 and 21, respectively. Beetles emerging from the seeds were mostly of the species *Acanthoscelides obtectus*, with *Zabrotes subfasciatus* only present in two plants, so both species were pooled for analyses. We found that damage treatment had an effect on the abundance of seed beetles ( $\chi_{\left(3\right)}^{2}$ =22.83; P<0.0001; **Figure 3B**). The number of seed insects in seeds from control plants was not significantly different from plants from the S-J treatment (Tukey’s *post-hoc* test: P=0.27), but was higher than in J and J-R treatment (Tukey’s *post-hoc* test: P=0.003 and P<0.001, respectively). When the number of seeds per plant was used as a covariate to explain insect abundance, its effect was highly significant ( $\chi_{\left(1\right)}^{2}$ =25.41; P<0.0001), but the effect of damage treatment was no longer important ( $\chi_{\left(3\right)}^{2}$ =0.79; P=0.85), with more insects emerging from plants that produced more seeds. The parasitism rate on beetles was in average 0.122 ± 0.038 and was not different among damage treatments ( $\chi_{\left(3\right)}^{2}$ =0.62; P=0.89). The structural equation model confirmed the association among variables and showed an association between number of leaves and number of seeds (**Figure 4**). Damage treatment affected the number of trifolia, however this path does not show an influence on number of seeds. Thus, the effect of treatment on number of seeds was independent from the number of trifolia. There is an indirect effect of treatment on number of seed beetles, mediated by number of seeds, with no direct effect of treatment on number of beetles.

## Discussion

The ability of a plant to recover from herbivore damage is crucial for its success in a natural environment. Plant ontogeny is known to play an important role in determining the compensation and defense responses against herbivory. While most studies on the effects of plant ontogeny on induced responses focus on isolated developmental stages, in nature, herbivores can attack throughout plant development at different points in time. Here we aimed to study the consequences of herbivory occurring at more than one ontogenetic stage with repeated or single damage events, on plant compensatory responses. This approach can give us information about the influence of early damage on the responses to subsequent damage events. We found that early damage (at the seedling and juvenile stages) results in better compensatory growth and seed output than late damage (at the juvenile stage alone, or together with damage at the reproductive stage). But early and repeated damage is still positive in terms of leaf and seed production when compared to a single damage event occurring at the juvenile stage. This indicates that damage at the seedling stage can enhance plant tolerance to future damage. Conversely, we did not find evidence that leaf damage induced defenses on seeds against attack by seed beetles. Infestation by beetles in seeds of damaged plants was not so different from infestation in seeds from undamaged control plants, regardless of the timing and number of damage events.

Plants damaged at the seedling stage compensated by producing more leaves than plants from the other damage treatments, while maintaining a similar leaf production compared to control plants. This suggests that early damage stimulated growth to compensate for the loss of photosynthetic area, as compared to plants damaged at later stages. A larger photosynthetic area as compared to plants damaged later in their development could provide enough resources to produce more seeds (Tiffin, 2000; Wise and Abrahamson, 2005). However, the SEM in the present study showed no association between number of trifolia and seed set, but a direct effect of damage treatment on the number of leaves, which indicates a change in resource allocation. A plausible explanation may be that when the apical meristem of seedlings was cut, it stimulated branching and this increased leaf and flower production (Tiffin, 2000). Interestingly, the number of branches was not affected by early damage, so that compensation was only for leaf production with no evidence of change in plant morphology in response to early herbivory. A meta-analysis on defense ontogeny (Barton and Koricheva, 2010) showed that tolerance does not generally change with ontogenetic stage. This could be an indicator that the ontogenetic patterns in tolerance are not consistent and depend on the plant system. If tolerance is an adaptation to the specific community and its interactions, then it could be expected that each plant species evolves compensatory responses well suited for their biotic environment (Pearse et al., 2017). However, this meta-analysis did not include studies that analyze tolerance to seedling damage. As seedlings have limited resources available from photosynthetic area or root reserves, they are expected to invest more in growth than in resistance strategies (Barton and Boege, 2017). Damage at the seedling stage can be particularly relevant, given the lack of resources in this early developmental stage (Barton and Hanley, 2013; Quintero and Bowers, 2013), any stressor could drastically alter resource allocation with consequences for the whole plant development. Thus, interactions at the seedling stage deserve more attention in studies of tolerance ontogeny.

Induced responses to damage can also be influenced by the plant’s exposure to previous events. For example, plants can be primed with stimuli such as egg oviposition, early herbivore damage or volatiles from damaged neighbors that prepare them for future attacks and increase the induction of defenses (Conrath et al., 2006; Heil and Kost, 2006; Hilker et al., 2016; Martinez-Medina et al., 2016). However, priming effects are not commonly linked to the responses in different ontogenetical stages. It is possible that when a stimulus occurs in an early ontogenetic stage of the plant, it could prime it to induce responses for later damage events. Thus, priming and ontogenetic trajectories can be linked by these early damage events. Yet, very few studies have examined the effect of an initial herbivory event at early ontogenetic stages on future plant compensatory responses. In wild radish, mechanical damage at the juvenile stage reduced seed output, while damage at a reproductive stage did not (Boege et al., 2007). However, when both types of damage were applied, seed output was similar to that in plants with only juvenile damage. As the specific physiological changes that take place to induce compensation need an external stimulus, it is possible that an early stimulus such as seedling damage, could prime the plant’s compensatory responses to future herbivory. While priming for future attacks is a phenomenon expected to occur in most plant species, it has been mainly tested for resistance and not for tolerance traits (Conrath et al., 2006; Hilker et al., 2016). In our study we show that seed output was higher for plants damaged as seedlings after repeated damage at the juvenile stage compared to plants damaged at only the juvenile stage or at the juvenile and reproductive stage, indicating that plants damaged at the seedling stage tolerated damage better than plants damaged at later stages. This, supports the existence of priming for tolerance in lima bean. Importantly, this is not an effect of only repeated damage, since plants damaged at the juvenile and reproductive stage did not compensate better than plants with one damage event at the juvenile stage. Alternatively, it is possible that damage at the seedling stage alters the plant’s resource allocation with an increased investment on growth and seed output, regardless of future damage.

Other types of priming stimuli for tolerance that are independent of damage such as application of oral secretion from herbivores or exposure to plant volatiles, can be tested. Induction of enhanced growth by volatiles has been shown for some plant species, including *Arabidopsis thaliana* (Shimola and Bidart, 2019), *Brassica* sp. (Pashalidou et al., 2020), lima bean (Freundlich et al., 2021) and *Medicago trunculata* (Maurya et al., 2022). In *Brassica*, volatiles emitted by plants infested with eggs of *Pieris brassicae* induce a higher reproductive output in undamaged exposed plants than in plants not exposed to volatiles. But when exposed plants were damaged by *P. brassicae* caterpillars, the reproductive output was similar to that of damaged plants not exposed to plant volatiles (Pashalidou et al., 2020). Thus, in this case tolerance to herbivory was not affected by exposure to eggs-infested plant volatiles. Tolerance can increase plant fitness in environments where herbivory is intense (Fornoni, 2011). Since it allows plants to reproduce despite herbivore damage, it is especially useful when herbivores are adapted to resistance traits (Best et al., 2008; Fornoni, 2011). Thus, compensatory responses associated with tolerance to herbivory play an important role in the establishment and maintenance of plant individuals and populations in communities with intense antagonistic interactions. Future studies should examine with more detail different tolerance priming stimuli and their outcome at varying herbivore pressure.

Induced resistance to herbivory at different ontogenetic stages is also an important component of plant defense. Here, our results show that plants damaged at the juvenile and reproductive stages had fewer seed insects than control plants, while plants damaged at the seedling stage had intermediate numbers of seed insects. However, this was driven by a density dependent effect of the number of seeds, with more seed insects present in plants with more seeds. Therefore, we did not detect an induction of seed defenses, which may suggest that this mechanism can vary with environmental conditions and the type of damage. This result could be also influenced by the nature of the damage (herbivores vs. mechanical damage). Induction of plant defenses have been shown to be influenced by direct cues from herbivores such as elicitors or microbes (Shikano et al., 2017), but also the type and amount of damage may play a role. For example, a single mechanical damage event did not induce herbivore associated plant volatiles in lima bean plants, but continuous mechanical damage can replicate the whole spectrum of volatiles as compared to plants damaged by herbivores (Mithöfer et al., 2005). Thus, it seems likely that the mechanical damage used in our study was not enough to induce defenses in the seeds as has been shown in studies using damage by herbivores in lima bean (Hernández-Cumplido et al., 2016; Bustos-Segura et al., 2020). Mechanical damage allowed us to control for the amount of damage among plants and between different time points which would not have been possible with herbivores in field conditions. Moreover, the amount of damage is particularly important for analyzing tolerance to herbivory (Muola et al., 2010). Previous studies on indirect defenses, plant volatiles (Heil and Silva Bueno, 2007) and extrafloral nectar (Blue et al., 2015), and on plant-plant communication (Moreira et al., 2016) have shown that mechanical damage induces plant responses in lima bean. However, the difference between the effects of mechanical and natural damage on lima bean seed defenses and tolerance has not been examined.

In synthesis, we provide evidence for the role of ontogeny in plant resource allocation which results in differential growth compensation and tolerance. We also show that frequency together with timing of damage will affect these responses, as early damage influenced the plant’s tolerance to future damage. Given that in nature, interactions with herbivores occur throughout the plant’s life, we emphasize the importance of analyzing the consequences of plant herbivory at multiple ontogenetic stages. Such an approach will increase our understanding of the factors associated with plant adaptations to a dynamic biotic environment.

## Data availability statement

The raw data supporting the conclusions of this article will be made available by the authors, without undue reservation.

## Author contributions

CB-S and BB: Conceptualization. CB-S and RG-S: Investigation. CB-S: Data curation and formal analysis. CB-S: Methodology. BB: Funding acquisition. CB-S: original draft preparation. CB-S and BB: review and editing. All authors contributed to the article and approved the submitted version.

## Funding

This research was financially supported by the Swiss National Science Foundation (Project No. 31003A_162860) awarded to BB.

## Acknowledgments

We thank the Universidad del Mar, campus Puerto Escondido for logistical support, particularly Dr. Jose Arcos and Alfredo Lopez-Rojas. We also thank Lucas Malacari and Yosra Chabaane for help in the field and Philippine Surer for her assistance with the seed analyses. The authors declare no conflict of interest.

## Conflict of interest

The authors declare that the research was conducted in the absence of any commercial or financial relationships that could be construed as a potential conflict of interest.

## Publisher’s note

All claims expressed in this article are solely those of the authors and do not necessarily represent those of their affiliated organizations, or those of the publisher, the editors and the reviewers. Any product that may be evaluated in this article, or claim that may be made by its manufacturer, is not guaranteed or endorsed by the publisher.
