# Supplementary material for: Early damage enhances compensatory responses to herbivory in wild lima bean
Source: Front Plant Sci. 2022 Nov 29;13:1037047. doi: 10.3389/fpls.2022.1037047 (PMC9745087; doi:10.3389/fpls.2022.1037047)
Supplement: Supplementary file 1 [file DataSheet_1.pdf]

Supplementary material

Early damage enhances compensatory responses to herbivory in wild lima bean

Carlos Bustos-Segura*, Raúl González-Salas, Betty Benrey

Laboratory of Evolutionary Entomology, Institute of Biology

***Correspondence:**

Carlos Bustos-Segura

bustossc@gmail.com

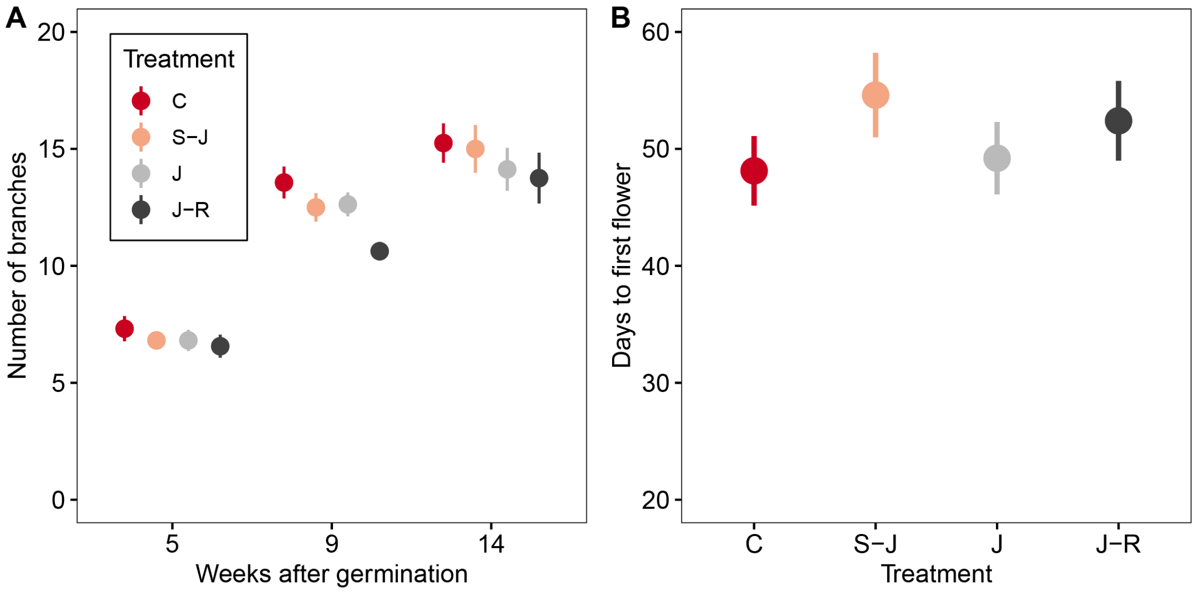

Figure S1. Effects of timing and frequency of damage on branches and time to flowering in wild lima bean plants. A) number of branches, B) days from germination to production of the first flower. Treatment groups were: mechanically undamaged control plants (C); plants damaged at the seedling and juvenile stage (S-J), only at the juvenile stage or at the juvenile stage and reproductive stage (J-R). No statistically significant differences were found among damage treatments within the same time point.

Table S1. Summary of the contrasts between damage treatments from generalized linear mixed models (Poisson distribution) on number of trifolia and seeds. Treatment groups were: mechanically undamaged control plants (C); plants damaged at the seedling and juvenile stage (S-J), only at the juvenile stage or at the juvenile stage and reproductive stage (J-R). Estimates are given on the log scale.

Contrast	Estimate	SE	z.ratio	p value	Cohen's d
**Trifolia number**					
C - (S-J)	0.0253	0.0387	0.655	0.9139	0.0253
C - J	0.1114	0.039	2.86	0.022	0.1114
C - (J-R)	0.0764	0.0388	1.966	0.2008	0.0764
(S-J) - J	0.0861	0.0391	2.201	0.123	0.0861
(S-J) - (J-R)	0.0511	0.039	1.309	0.5572	0.0511
J - (J-R)	-0.035	0.0393	-0.892	0.8088	-0.035
**Seed number**					
C - (S-J)	0.46926	0.0551	8.511	<.0001	0.46926
C - J	0.69875	0.0582	12.016	<.0001	0.69875
C - (J-R)	0.6928	0.0584	11.863	<.0001	0.6928
(S-J) - J	0.22949	0.0651	3.528	0.0024	0.22949
(S-J) - (J-R)	0.22354	0.0653	3.424	0.0035	0.22354
J - (J-R)	-0.00595	0.0678	-0.088	0.9998	-0.00595
J-(J-R)	-0.00595	0.0678	-0.088	0.9998	-0.00595
